# Effects of mitochondrial gene mutation 8923 (A→G) on inflammatory indicators and blood pressure after acute high-altitude exposure

**DOI:** 10.3389/fphys.2026.1777284

**Published:** 2026-02-26

**Authors:** Minglei Zhang, Ruizhe Li, Yining Liu, Zongbin Li

**Affiliations:** 1 Senior Department of Cardiology, The Sixth Medical Center of Chinese PLA General Hospital, Beijing, China; 2 School of Medicine, Nankai University, Tianjin, China; 3 Chinese PLA Medical College, Beijing, China

**Keywords:** blood pressure, clinical, gene mutation, inflammatory indicators, mitochondrial

## Abstract

**Background:**

In recent years, an increasing number of individuals have traveled to or resided in plateau regions for various reasons. The hypobaric hypoxia characteristics of plateau environments represents a key risk factor for acute mountain sickness (AMS). The pathogenesis of AMS remains incompletely understood. The present study aimed to explore the association between the A8923G point mutation in the mitochondrial MT-ATP6 gene and AMS.

**Methods:**

We enrolled 84 healthy adult male volunteers who traveled together from the plain (Beijing, <100 m) to a 4000-m plateau in <40 h. Peripheral venous blood was collected for related tests; volunteers also underwent ambulatory blood pressure/electrocardiography monitoring. We analyzed these physiological indicators to examine the association between the MT-ATP6 A8923G mutation and AMS.

**Results:**

After acute high-altitude exposure, the mutation group had lower C-reactive protein (CRP) [0.04 (0.03,0.04) vs. 0.07 (0.03.0.13), *P* = 0.045] and higher high-density lipoprotein cholesterol (HDL-C) [1.5 ± 0.4 vs. 1.3 ± 0.3, *P* = 0.021] than the non-mutation group, plus lower 24-h and nocturnal mean systolic blood pressure (SBP) (all *P* < 0.05), with significant intergroup differences.

**Conclusion:**

The A8923G point mutation acts as a protective locus against AMS. It was associated with lower high-altitude SBP (more pronounced at night), reduced CRP and elevated HDL-C, possibly by inhibiting inflammation and enhancing blood pressure regulation post high-altitude exposure.

## Introduction

Statistics show that tens of millions of people enter high-altitude regions (≥2,500 m) annually for work, sports, training, and other purposes. For individuals born and raised in lowland areas, a major concern is the high risk of acute mountain sickness (AMS) after acute high-altitude exposure. Previous studies indicate that residing below 1,500 m is a significant independent risk factor for AMS (Gao et al., 2024). In recent years, research on the genetic pathogenesis of AMS has advanced rapidly, covering adaptive and susceptibility genes. Besides nuclear genes research, notable progress has been made in mitochondrial gene studies.

Acute altitude sickness is a common disorder unique to high-altitude regions. It referrs to various pathological reactions that occur hours to days after exposure to the hypobaric hypoxic environment at elevations above 2,500 m (Basnyat and Murdoch, 2003; Kayser and Verges, 2013; Meier et al., 2017). AMS is the most prevalent form, while other types include high-altitude pulmonary edema (HAPE) and high-altitude cerebral edema (HACE). AMS is generally a self-limited condition with non-specific clinical manifestations. Its four core symptoms are headache, fatigue, gastrointestinal symptoms, and dizziness, with headache being the most prominent. The incidence of AMS is closely linked to ascent rate, altitude, hypoxic adaptability, and individual susceptibility (Gatterer et al., 2024). At 2,500 m, the incidence is approximately 10%. It exceeds 25% above 3,500 m and affects over 50% of individuals at 6,000 m (Gao et al., 2024; Gatterer et al., 2024; Bärtsch and Swenson, 2013). As a self-limited disorder, AMS symptoms typically resolve with oxygen therapy, rest, or descent. However, approximately 1% of patients may progress to life-threatening HAPE or HACE. Therefore, research into the pathophysiological mechanisms of AMS has received considerable attention (McGowan et al., 2025), but the underlying genetic factors remain poorly understood. Although multiple studies have investigated the correlation between mitochondrial gene mutations and AMS (Li et al., 2016; Li, 2013; Luo et al., 2011; Li, 2011), reports on the association between specific point mutations and AMS are limited. We previously identified protective mitochondrial DNA mutations against AMS, including the MT-ATP6 A8923G variant (Li et al., 2022). However, its functional mechanism has not been fully elucidated. This study further analyzes the potential targets of the A8923G point mutation using existing data.

Currently, the diagnosis of AMS relies mainly on its typical clinical manifestations; to date, there are no specific physiological indicators for definitive diagnosis (Richalet et al., 2024). If we can screen for AMS risk using certain test indicators and assess disease susceptibility before individuals enter high-altitude regions, we can effectively reduce AMS incidence. This can be achieved by excluding high-risk groups from high-altitude areas, conducting pre-exposure training, or implementing pharmacological interventions. In our study, we performed whole-genome sequencing on participants and collected physiological indicator data (e.g., ambulatory blood pressure, electrolytes) from all volunteers. We further compared physiological differences between volunteers with and without the mutation after acute high-altitude exposure, and verified the correlation between the mitochondrial MT-ATP6 gene A8923G point mutation and AMS in high-altitude environments. By investigating the potential targets of this mitochondrial gene mutation, we aim to provide new insights for the early screening of high-risk AMS populations and disease prevention.

## Materials and methods

### Research objects

#### Ethical statement

Volunteers were recruited through open enrollment. This study was conducted in accordance with the Declaration of Helsinki and approved by the Ethics Committee of Chinese PLA General Hospital (Approval No.: S2025-801-01). All volunteers signed informed consent forms after being fully informed of the study details. Throughout the experiment, experienced clinicians were on standby, and standard emergency medications were prepared.

#### General information

After screening, 84 adult male volunteers were finally enrolled in the study.

Inclusion criteria: (1) Male gender; (2) Aged 18–35 years; (3) Han ethnicity, born and raised in plain areas with no prior high-altitude exposure.

Exclusion criteria: (1) History of cardiovascular disease, respiratory disease, and other chronic illnesses; (2) History of primary headache disorders or other relevant conditions; (3) Presence of digestive system disorders or malignancies, or symptoms such as vomiting; (4) Use of medications with a clear preventive effect on acute altitude sickness; (5) Current participation in other clinical trials.

### Mutational analysis

Genomic DNA was isolated from volunteers’ whole blood using Genomic DNA Isolation Kits (Thermo Fisher Scientific, Minneapolis Massachusetts, MA). The mitochondrial genome was enriched and purified by long-range polymerase chain reaction (PCR) using human mitochondrial genome-specific primers. The primer sequences are as follows: Forward 1, 5′-GACGGGCTCACATCACCCCATAA-3′, Reverse1, 5′-GCGTACGGCCAGGGCTATTGGT-3’; Forward 2, 5′-GGTGGCTGGCACGAAATTGACC-3′, Reverse 2, 5′-GCCACAACTAACCTCCTCGGACTCCT-3’. Sequencing libraries were prepared according to the protocols of the Sure Select QXT Library Prep Kit (Agilent, California, CA). Sequences obtained from sample sequencing were compared with the revised Cambridge Reference Sequence (rCRS, GenBank NC_012920.1) in the database. GATK analysis software was used to count the read counts and total data output of sequencing fragments, and to evaluate sequencing depth, coverage, and uniformity. Each cycle of all sequencing reads was monitored to assess sample and sequencing quality. Sequencing quality analysis showed that 99.14% of reads had a sequencing quality score greater than Q30.

### Main experimental instruments and materials

The following instruments were used: ambulatory blood pressure monitor (Suntech, USA), pulse oximeter (Tyco, USA), and 12-lead simultaneous ambulatory electrocardiogram system (Shiji Jinke, China).

The ambulatory blood pressure monitor was set to take measurements every 30 min, with the nighttime sleep period set from 22:00 to 06:00 the next morning.

### Baseline data collection

At enrollment, all volunteers underwent baseline data assessment, including height, weight, age, diastolic blood pressure (DBP), systolic blood pressure (SBP), heart rate, 24-h ambulatory electrocardiogram, ambulatory blood pressure monitoring, and other demographic and clinical data. Body mass index (BMI) was also calculated. Peripheral venous blood samples were collected for blood biochemical analysis and mitochondrial gene extraction. These results served as baseline data for enrollment. Volunteers with significant abnormalities in baseline indicators were excluded from subsequent experiments.

### High-altitude experiments

Upon recruitment, we collected demographic data (age, height, weight, and BMI) from all volunteers. Baseline measurements, including complete blood count (CBC), blood biochemistry, blood pressure, and electrocardiography (ECG), were obtained in Beijing (a plain region). After resting for several hours at 2800 m, all volunteers were transported by bus to the final destination (4000 m) within 3 h, where monitoring was terminated. Upon arrival at 4000 m, a treadmill exercise test was conducted as the first procedure (Ahluwalia and Underwood, 2024; Hackett and Oelz, 1992; Roach et al., 2018). Within the following 2 h, peripheral oxygen saturation (SpO_2_) was measured, and blood samples were collected. These samples were analyzed for hemoglobin concentration, white blood cell count and differential, platelet count, liver and kidney function, serum electrolytes, and myocardial injury markers. All volunteers completed a treadmill exercise test within 6 h. Resting baseline physiological indicators (SpO_2_, blood pressure, and heart rate) were recorded before exercise. During exercise, heart rate, blood pressure, and SpO_2_ were monitored every minute until 3 min afte exercise cessation.

### Statistical methods

Statistical analyses were performed using IBM SPSS Statistics version 25.0 (IBM Corp., Armonk, NY, USA). The Shapiro-Wilk test was used to assess the normality of all quantitative variables, and the Levene test was applied to check for homogeneity of variance. Normally distributed continuous data are presented as the mean ± standard deviation $\left(\bar{x}\pm s\right)$ . Non-normally distributed quantitative data are expressed as the median and interquartile range [ $M\left(P_{25},P_{75}\right)$ ]. Qualitative data were expressed as counts (percentage, *%*). Intergroup comparisons of normally distributed quantitative data were conducted using the independent samples t-test, and intragroup comparisons were performed with the paired samples t-test. For non-normally distributed quantitative data, intergroup comparisons were analyzed by Mann-Whitney U test, and intragroup comparisons were carried out using the Wilcoxon signed-rank test for paired samples. Qualitative data were compared using the chi-square test or exact probability method. The Bonferroni correction was applied for *post hoc* pairwise comparisons in multiple-group analyses and for pairwise comparisons of categorical variables in chi-square tests to control the type I error associated with multiple comparisons. A *P* value < 0.05 was considered statistically significant.

## Results

### The development of AMS

As shown in Table 1, only two of the 15 volunteers with the mitochondrial DNA MT-ATP6 gene A8923G point mutation developed acute mountain sickness (AMS), accounting for 13.3% (2/15). In contrast, 32 of the remaining 69 volunteers developed AMS, representing 46.4% (32/69). Pearson $\chi^{2}$ test showed a statistically significant difference between the two groups (*χ^2^* = 5.58, *P* = 0.018). Mutation carriers had an 82% lower risk of developing AMS (*OR* = 0.18, 95% *CI*: 0.04–0.85).

**TABLE 1:** Fourfold table of MT-ATP6 A8923G mutation status and AMS occurrence.

	AMS− (no acute mountain sickness)	AMS+ (acute mountain sickness)	Row total
Mutation group	13	2	15
Non-mutation group	37	32	69
Column total	50	34	84

### Mutation analysis of the MT-ATP6 gene A8923G locus

Our previous study detailed mitochondrial gene sequencing in 84 volunteers (Li et al., 2022). After comparing next-generation sequence (NGS) results with the reference sequence, we identified 3,923 mutation loci in the gene coding region, involving 37 mitochondrial genome genes. A8923G is a locus in the mitochondrial DNA (mtDNA) MT-ATP6 gene, which encodes the ATP6 subunit of mitochondrial complex V. This mutation causes an amino acid change from threonine to alanine. It has been reported in Mitomap but lacks clear disease association records. Single-locus comparisons between AMS-positive (AMS+) and AMS-negative (AMS-) groups showed statistically significant differences in 21 mutation loci, including the A8923G locus (present by both groups) located in the complex V subunit. Of the 84 enrolled volunteers, 15 carried the A8923G mutation. We performed functional predictions of this mutation using three online tools—Mutation Taster, Polyphen, and SIFI—with results of Polymorphism, Tolerated, and Probably Damaging, respectively. According to the species conservation index calculated via the Mitool website, 13 out of 21 mutation loci had a conservation index ≥0.80, among which the MT-ATP6 gene A8923G locus had a conservation index of 1.

### Physiological analysis

#### Baseline analysis at enrollment

The A8923G mutation is present in both AMS+ and AMS- groups. Among the 84 enrolled volunteers, 15 carried the A8923G mutation, accounting for 17.9% of the total cohort.

No statistically significant differences in age, height, weight, and BMI were observed between the two volunteer groups at enrollment. Under plain-region conditions, no statistically significant differences in routine blood test results, renal function, liver function, blood lipid profiles, blood pressure, or other relevant clinical indicators were detected between the two groups at enrollment (Table 2).

**TABLE 2:** Basic characteristics of volunteers stratified by MT-ATP6 gene A8923G mutation status.

variable	Mutation group (n = 15)	Non-mutation group (n = 69)	*P* value
Age, years	26 (23, 28)	25 (24, 27)	0.873
Height, cm	175.27 ± 5.13	174.93 ± 4.61	0.801
Weight, kg	66 (65, 70)	66 (65, 71)	0.209
BMI, kg/m^2^	21.97 (20.15, 24.16)	23.12 (21.43, 24.73)	0.169
White blood cell count, ×10^9^/L	6.31 (5.62, 6.92)	6.65 (5.60, 8.06)	0.546
C-reactive protein, mg/dl	0.07 (0.05, 0.09)	0.10 (0.06, 0.14)	0.111
HDL-C, mmol/L	1.5 ± 0.3	1.4 ± 0.3	0.672
24-h meanSBP, mmHg	113.48 ± 6.2	114.1 ± 7.0	0.602
Nocturnal mean SBP, mmHg	105.0 (100.5, 106.0)	106.0 (101.9, 110.6)	0.371
Nocturnal SBP load, %	0 (0, 12.5)	10.6 (0, 12.5)	0.801
MET, METs	10.1 (10.1, 10.1)	10.1 (10.1, 10.1)	0.707

Data with normal distribution are presented as mean ± standard deviation $\left(\bar{x}\pm s\right)$ . Data with non-normal distribution are presented as median [interquartile range, $M\left(P_{25},P_{75}\right)$ ]. Between-group comparisons were performed using independent-samples t-test or Mann-Whitney U test, as appropriate.

### Analysis of routine blood and biochemical test results at high-altitude

After acute high-altitude exposure, C-reactive protein (CRP) levels were significantly lower in volunteers carrying the A8923G mutation than that in the non-mutation group [0.04 (0.03,0.04) vs. 0.07 (0.03.0.13), *P* = 0.045], while high-density lipoprotein cholesterol (HDL-C) levels were significantly higher [1.5 ± 0.4 vs. 1.3 ± 0.3, *P* = 0.021]. Both differences were statistically significant (Table 3). No statistically significant differences were observed between the two groups in hemoglobin, platelet count, low-density lipoprotein cholesterol (LDL-C), electrolytes, or other relevant indicators. Notably, these indicators showed no statistical differences between the two groups under plain-region conditions.

**TABLE 3:** Routine blood and biochemistry profiles of the two volunteer groups at high-altitude.

variable	Mutation group (n = 15)	Non-mutation group (n = 69)	*P* value
White blood cell count, ×10^9^/L	7.9 (6.6, 8.9)	8.0 (6.7, 9.7)	0.503
Red blood cell count, ×10^12^/L	5.29 (5.09, 5.60)	5.26 (5.02, 5.44)	0.470
Hemoglobin, g/L	160.0 (155.0, 170.0)	163.0 (157.5, 167.5)	0.799
Platelet count, ×10^9^/L	227.0 (193.0, 296.0)	231.0 (203.0, 257.5)	0.615
C-reactive protein, mg/dl	0.04 (0.03, 0.04)	0.07 (0.03.0.13)	0.045
Triglycerides, μmol/L	1.3 (0.8, 1.8)	1.5 (1.0, 1.9)	0.680
LDL-C, mmol/L	2.6 ± 0.6	2.6 ± 0.7	0.920
Sodium, mmol/L	144.0 (143.0, 145.0)	143.0 (142.0, 145.0)	0.468
Aspartate aminotransferase, U/L	15.0 (14.0, 18.0)	17.0 (15.0, 20.0)	0.183
Creatine kinas, U/L	117.8 (114.9, 201.4)	131.8 (96.4, 157.9)	0.612
γ-glutamyltransferase, U/L	17.0 (14.0, 24.0)	17.0 (14.0, 25.5)	0.472
Cardiac troponin T, ng/ml	0.005 (0.003, 0.006)	0.004 (0.003, 0.005)	0.511
Total cholesterol, umol/L	4.4 (4.1, 5.0)	4.3 (3.9, 4.8)	0.561
HDL-C, mmol/L	1.5 ± 0.4	1.3 ± 0.3	0.021
Potassium, mmol/L	3.9 (3.8, 4.2)	4.1 (3.9, 4.3)	0.383
Chloride, mmol/L	102.1 (100.9, 103.1)	102.5 (101.2, 103.8)	0.300
Alanine aminotransferase, U/L	16.0 (12.0, 20.0)	17.0 (14.0, 26.0)	0.292

Data with normal distribution are presented as mean ± standard deviation $\left(\bar{x}\pm s\right)$ . Data with non-normal distribution are presented as median [interquartile range, $M\left(P_{25},P_{75}\right)$ ]. Between-group comparisons were performed using independent-samples t-test or Mann-Whitney U test, as appropriate.

### Analysis of ambulatory electrocardiogram and ambulatory blood pressure after acute high-altitude exposure

After acute high-altitude exposure, the A8923G mutation group had significantly lower levels of 24-h mean systolic blood pressure (115.8 ± 6.7 vs. 120.5 ± 8.0, *P* = 0.039), nocturnal mean systolic blood pressure (107.9 ± 6.9 vs. 113.4 ± 7.4, *P* = 0.011), and nocturnal systolic blood pressure load [18.8 (0,25.0) vs. 29.4 (12.5,47.1), *P* = 0.022] compared with the non-mutation group. These differences were statistically significant (Table 4). No statistically significant differences between the two groups were observed in total heart rate, mean heart rate, 24-h mean diastolic blood pressure, nocturnal mean diastolic blood pressure, 24-h mean pulse rate, or other related indicators. Identified arrhythmia types included atrial premature beats, atrial escape beats, non-sustained atrial tachycardia, junctional premature beats, and ventricular premature beats. The incidence of these arrhythmias did not differ significantly between the two groups.

**TABLE 4:** Ambulatory blood pressure and ambulatory electrocardiogram data of the two volunteer groups at high altitude.

variable	Mutation group (n = 15)	Non-mutation group (n = 69)	*P* value
Total heart rate, bpm	68,018 (61292, 79,706)	75,704 (61166, 82,515)	0.417
Mean heart rate, bpm	69 (56, 81)	56 (47, 77)	0.111
HRV triangular index	36.8 (29.3, 44.2)	35.9 (32.4, 53.1)	0.424
Atrial premature beats, %	10 (66.7%)	45 (65.2%)	0.832
Atrial escape beats, %	0	2 (2.9%)	0.505
Non-sustained atrial tachycardia, %	0	1 (1.4%)	0.639
Junctional premature beats, %	0	1 (1.4%)	0.639
Junctional escape beats, %	0	0	-
Ventricular premature beats, %	4 (26.7%)	15 (21.7%)	0.679
Ventricular escape beats, %	0	1 (1.4%)	0.639
Non-sustained ventricular tachycardia, %	0	0	-
24-h mean systolic blood pressure, mmHg	115.8 ± 6.7	120.5 ± 8.0	0.039
24-h mean diastolic blood pressure, mmHg	73.7 ± 6.8	75.0 ± 6.8	0.495
Nocturnal mean systolic blood pressure, mmHg	107.9 ± 6.9	113.4 ± 7.4	0.011
Nocturnal mean diastolic blood pressure, mmHg	65.7 ± 6.8	68.0 ± 6.0	0.204
Nocturnal systolic blood pressure load, mmHg	18.8 (0,25.0)	29.4 (12.5, 47.1)	0.022
Nocturnal diastolic blood pressure load, mmHg	5.9 (0,12.5)	11.8 (0,19.4)	0.398
24-h mean pulse rate, bpm	80.0 ± 8.5	80.1 ± 9.0	0.784

Data with normal distribution are presented as mean ± standard deviation $\left(\bar{x}\pm s\right)$ . Data with non-normal distribution are presented as median [interquartile range, $M\left(P_{25},P_{75}\right)$ ]. Between-group comparisons were performed using independent-samples t-test or Mann-Whitney U test, as appropriate.

### Analysis high-altitude treadmill exercise test results

As shown in Table 5, after acute high-altitude exposure, the metabolic equivalent of task (MET) level was slightly higher in volunteers carrying the A8923G mutation than that in the non-mutation group [10.0 (9.8,10.2) vs. 10.0 (7.5,10.0), *P* = 0.043], and this difference was statistically significant. No statistically significant differences in heart rate, systolic blood pressure, Saturation of Peripheral Oxygen (SpO_2_), or other relevant indicators were observed between the two groups before and after the treadmill exercise test.

**TABLE 5:** High-altitude treadmill exercise test data of the two volunteer groups.

variable	Mutation group (n = 15)	Non-mutation group (n = 69)	*P* value
Pre-exercise SpO_2_, %	81 ± 7	82 ± 6	0.577
Post-exercise SpO_2_, bpm	72 (68, 75)	74 (70, 78)	0.192
Pre-exercise heart rate, bpm	94 ± 17	92 ± 14	0.638
Post-exercise heart rate, bpm	146 (128, 150)	144 (134, 155)	0.312
Exercise time, s	444 (420, 480)	425 (383, 479)	0.231
MET, METs	10.0 (9.8, 10.2)	10.0 (7.5, 10.0)	0.043
3 min post-exercise SBP, mmHg	143 (127, 152)	146 (136, 156)	0.326
3 min post-exercse DBP, mmHg	71 (67, 73)	71 (63, 78)	0.991
Mean heart rate during the first 3 min post-exercise, bpm	122 ± 23	115 ± 24	0.330

Data with normal distribution are presented as mean ± standard deviation $\left(\bar{x}\pm s\right)$ . Data with non-normal distribution are presented as median [interquartile range, $M\left(P_{25},P_{75}\right)$ ]. Between-group comparisons were performed using independent-samples t-test or Mann-Whitney U test, as appropriate.

## Discussion

In our previous study, we used the LLQS as the diagnostic criterion and performed mitochondrial whole-genome sequencing on volunteers’ peripheral blood samples. We confirmed that the mitochondrial DNA (mtDNA) MT-ATP6 gene 8923 A>G point mutation acts as a protective factor against AMS. However, its underlying mechanism of action has not been further elucidated. Comparative analysis of various biochemical indicators and auxiliary examination results between A8923G mutation carriers and non-carriers revealed several significant differences and notable patterns.

In the present study, we found that the A8923G mutation is associated with the specific regulation of systemic inflammatory responses and lipid metabolism in humans after acute high-altitude exposure. Carriers of this mutation had significantly lower CRP levels and notably higher HDL-C levels (Table 3). However, no statistically significant differences in these indices were observed between the two groups under plainland conditions (Table 2). These findings suggest that the A8923G mutation may participate in the body’s adaptive regulation in response to acute high-altitude hypoxic stress by modulating inflammatory homeostasis and lipid metabolism. This provides novel phenotypic evidence for elucidating the genetic regulatory mechanisms underlying high-altitude hypoxic adaptation. Acute high-altitude exposure can trigger a series of physiological and even pathological responses in the human body due to its unique environmental characteristics. As atmospheric pressure decreases, hypoxia becomes the most critical factor (Richalet et al., 2024). As a classic systemic inflammatory marker of the acute-phase response, changes in CRP levels directly reflect the degree of inflammatory activation in the body in response to hypoxic stress. The immune system’s dynamic regulation is a highly sophisticated process. Previous research shows that rapid ascent to high altitudes within hours can cause severe adverse physiological effects on the human immune system, potentially due to acute hypoxia and immune cell damage (Yan et al., 2024; Thake et al., 2004). The idea that hypoxia triggers the body’s inflammatory response is widely validated (Kammerer et al., 2020; Mirrakhimov et al., 1989). During acute high-altitude exposure, hypoxic stimulation can trigger vascular endothelial and systemic inflammatory responses. Elevated CRP levels are one of the core manifestations of such stress-induced inflammation. Excessive inflammatory activation exacerbates hypoxia-induced tissue damage and vascular endothelial dysfunction, thereby impairing the ability to adapt to high-altitude hypoxia. In the present study, CRP levels were significantly lower in mutation carriers than in the non-mutation group. This suggests that the A8923G mutation may reduce systemic inflammatory injury under high-altitude exposure by inhibiting the excessive activation of hypoxia-induced inflammatory. This phenotypic trait represents a potential protective adaptive feature of the human body in coping with high-altitude hypoxia. It also provides mechanistic insights at the inflammatory level for the enhanced tolerance to acute high-altitude hypoxia observed in A8923G mutation carriers.

HDL-C exerts anti-inflammatory and antioxidant effects, acting as a core biomarker with multiple protective properties. It not only mediates reverse cholesterol transport in peripheral tissues and inhibits atherosclerosis development but also alleviates hypoxic stress-induced vascular endothelial injury and microcirculatory dysfunction via antioxidant, anti-inflammatory, and endothelial protective actions. Vascular endothelial integrity is pivotal for maintaining tissue oxygen delivery and cardiovascular homeostasis in the high-altitude hypoxic environment. In the present study, A8923G mutation carriers maintained significantly higher HDL-C levels after acute high-altitude exposure, indicating that this mutation positively regulates lipid metabolic homeostasis under hypoxic stress. By enhancing HDL-C–mediated protection, the mutation strengthens the body’s cardiovascular adaptive capacity to high-altitude hypoxia and mitigates hypoxemia-induced cardiovascular damage. Together with reduced CRP levels, this creates a synergistic protective effect across inflammatory and lipid metabolic pathways, conferring mutation carriers a superior physiological adaptive state during acute high-altitude exposure.

Elevated white blood cell counts in AMS patients may linked to systemic inflammatory response syndrome (SIRS). Hypoxia promotes microvascular reactions, induces angiogenesis, and causes leukocyte migration and increased vascular permeability (Zhou et al., 2007; Gonzalez and Wood, 2001; Hennis et al., 2010; Mirrakhimov et al., 1989; Thake et al., 2004; Julian et al., 2011; Boos et al., 2016; Bailey et al., 2013; Irarrázaval et al., 2017; Bailey et al., 2009) This complex inflammatory response may be associated with neutrophil activation, indicating that individuals with elevated CRP levels have a higher risk of developing AMS after acute high-altitude exposure. Measuring HDL-C levels may provide insights for screening high-risk populations for AMS. Overall, these findings highlight the importance of understanding and targeting the body’s inflammatory response to high-altitude environments to improve adaptation and reduce potential risks.

Although inflammatory processes and vascular responses are regulated by multiple biological pathways, changes in a single inflammatory indicator cannot be fully attributed to the complex pathological mechanisms of AMS. In the present study, we only observed the association between the A8923G mutation and the CRP/HDL-C phenotypes after acute high-altitude exposure, its specific molecular regulatory mechanisms require further validation.

Another key finding was that after acute high-altitude exposure, the nocturnal mean systolic blood pressure (SBP) and 24-h mean SBP in the A8923G mutation group were significantly lower than those in the non-mutation group, with a mean difference exceeding 5 mmHg. Additionally, the two groups differed statistically in nocturnal SBP burden, with lower values observed in the A8923G mutation group (see Table 4). This observation is consistent with the blood pressure changes—primarily in SBP—seen in high-altitude environments as described in the book *High Altitude Diseases*. These results indicate that the A8923G mutation is closely associated with the regulation of systemic blood pressure homeostasis following acute high-altitude exposure. This finding further indicates that the A8923G mutation exerts a cardiovascular protective effect during acute high-altitude hypoxic adaptation by inhibiting hypoxia-induced abnormal systolic blood pressure elevation and improving nocturnal blood pressure regulatory rhythm (Naeije, 2010). During acute high-altitude exposure, hypoxic stimulation triggers a compensatory blood pressure increase via multiple pathways, including activation of the sympathetic nervous system-renin-angiotensin-aldosterone system (SNS-RAS), vascular endothelial dysfunction, and increased peripheral vascular resistance—all typical cardiovascular stress responses to hypoxia (Parati et al., 2014; Halliwill and Minson, 1985; Bilo et al., 2012). Notably, rapid ascent to high altitudes can elevate nighttime blood pressure, and nocturnal systolic blood pressure load—a key biomarker of abnormal blood pressure elevation—indicates prolonged vascular exposure to high pressure. This not only exacerbates vascular endothelial injury and microcirculatory dysfunction under hypoxia but also increases the risk of high-altitude hypertension (Bilo et al., 2019). In contrast, A8923G mutation carriers showed significantly lower nocturnal systolic blood pressure and associated load. This suggests the mutation protects against AMS by effectively inhibiting excessive hypoxia-induced sympathetic activation, mitigating SNS-RAS pathway hyperactivity, and reducing sustained peripheral vasoconstriction—thus preserving the normal dipper-like blood pressure rhythm at rest and alleviating vascular pressure load (Lang et al., 2016; Naeije, 2010; Chen et al., 2021).

Meanwhile, the significant reduction in 24-h mean systolic blood pressure confirms that the A8923G mutation’s blood pressure regulation under high-altitude hypoxia is not limited to the nocturnal resting phase, but rather provides sustained protection of global blood pressure homeostasis. This regulatory effect allows A8923G mutation carriers to avoid excessive compensatory blood pressure elevation during acute high-altitude exposure and reduces the overall stress load on the cardiovascular system. Additionally, the differences in nocturnal blood pressure-related indices are more pronounced than those in 24-h mean blood pressure, highlighting that the mutation’s modulation of blood pressure regulatory rhythm during rest is its core advantage. This trait is physiologically important for reducing the risk of cardiovascular adverse events under high-altitude hypoxia.

Furthermore, this study found that the A8923G mutation is closely associated with maintaining systemic aerobic metabolic capacity following acute high-altitude exposure. Although metabolic equivalent (MET) levels were numerically slightly higher in mutation carriers than in non-carriers, the difference was statistically significant: MET values in the mutation group were highly concentrated at high levels, while the non-mutation group showed a marked interindividual decline. This suggests the mutation effectively inhibits the reduction in aerobic metabolic capacity induced by acute high-altitude hypoxic stress, a crucial functional adaptive response to high-altitude hypoxia. It also confirms the mutation’s association with high-altitude hypoxic adaptation from a global metabolic function perspective.

Together with our previous findings—reduced CRP levels, elevated HDL-C levels and improved blood pressure homeostasis in A8923G mutation carriers—this study demonstrates that the mutation’s regulation of MET reflects multidimensional synergistic protection, rather than a single-pathway effect. This synergy is underpinned by three key mechanisms: inflammation inhibition, optimized lipid metabolism, and improved blood pressure homeostasis. Mutation-mediated reduction in systemic inflammation alleviates hypoxia-induced injury to vascular endothelial cells and cardiac muscle, preserving mitochondrial oxidative phosphorylation function. Concurrently, elevated HDL-C levels enhance antioxidant defenses and improve vascular endothelial vasomotor function, optimizing oxygen and nutrient delivery to peripheral tissues. Furthermore, the reduction in 24-h and nocturnal systolic blood pressure decreases myocardial oxygen consumption and peripheral circulatory load. This allows the body to maximize the efficiency of oxygen uptake, transport, and utilization under hypoxic conditions, ultimately safeguarding aerobic metabolic stability as reflected by preserved MET levels. These findings confirm that the A8923G mutation exerts multi-targeted, multi-level regulatory effects on high-altitude hypoxic adaptation: from core inflammatory and lipid metabolic processes, to cardiovascular homeostasis, and ultimately to overall systemic metabolic and functional adaptation.

### Limitations

In the present study, we only observed the association between the A8923G mutation and various physiological and biochemical indicators; its specific molecular regulatory mechanisms require further in-depth verification and elucidation. Due to the unique high-altitude environment and the incidence of AMS, this study included a total of 84 volunteers, with only 15 in the A8923G mutation group. This relatively small sample size—especially in the mutation subgroup—may have affected the stability of the statistical results. We did not perform subgroup analysis on volunteers who developed AMS, subsequent studies with an expanded sample size are needed to validate the above conclusions. Additionally, our study only included young Han Chinese males. In the future, it will be necessary to expand the sample size to include subjects of different genders, ethnicities, and age groups. Multi-center, large-sample validation studies should also be conducted to further verify the generalizability of our conclusions.

## Conclusion

The A8923G point mutation in the mitochondrial MT-ATP6 gene acts as a protective factor against AMS. After acute high-altitude exposure, mutation carriers exhibited significantly lower systolic blood pressure (particularly at night), reduced C-reactive protein levels and white blood cell counts, higher HDL-C levels, and stably elevated metabolic equivalent (MET) levels compared with non-carriers. These changes indicate that the mutation exerts a synergistic protective effect by suppressing excessive inflammatory responses, enhancing blood pressure regulation, optimizing lipid metabolism, and preserving aerobic metabolic function under hypoxic stress. This finding identifies key genetic determinants of AMS susceptibility and provides critical insights for developing personalized AMS prevention and intervention strategies for high-altitude populations. The distinct protective phenotypes associated with this mutation further highlight the critical role of mitochondrial genetic variation in mediating the body’s adaptive response to high-altitude hypoxia. Specifically, the MT-ATP6 gene-related regulatory effects maintain cardiovascular homeostasis and systemic metabolic efficiency while mitigating tissue damage induced by hypoxic stress. In conclusion, this study advances the understanding of the genetic mechanisms underlying high-altitude adaptation and establishes a preliminary foundation for subsequent mechanistic research on mitochondrial genes in regulating hypoxia tolerance.

## Data Availability

The data analyzed in this study is subject to the following licenses/restrictions: The participating individuals have explicitly withheld consent for public release of their full sequencing data. Requests to access these datasets should be directed to Zongbin Li, Lizww@163.com.
