# Correction: Effects of mitochondrial gene mutation 8923 (A→G) on inflammatory indicators and blood pressure after acute high-altitude exposure

**DOI:** 10.3389/fphys.2026.1828449

**Published:** 2026-05-06

**Authors:** Minglei Zhang, Ruizhe Li, Yining Liu, Zongbin Li

**Affiliations:** 1 Senior Department of Cardiology, The Sixth Medical Center of Chinese PLA General Hospital, Beijing, China; 2 Nankai University School of Medicine, Tianjin, China; 3 Chinese PLA Medical College, Beijing, China

**Keywords:** blood pressure, clinical, gene mutation, inflammatory indicators, mitochondrial

There was a mistake in **Table 1** as published. The patient numbers for AMS- and AMS+ in the “Non-mutation group” row—37 and 32—were accidentally swapped. The corrected **Table 1** appears below.

**Table 1:** Fourfold table of MT-ATP6 A8923G mutation status and AMS occurrence.

	AMS- (no acute mountain sickness)	AMS+ (acute mountain sickness)	Row total
Mutation group	13	2	15
Non-mutation group	37	32	69
Column Total	50	34	84

The data errors in **Table 1** also affected the corresponding results sections, which we have corrected.

A correction has been made to the section **Results**, *The Development of AMS*, Paragraph 1:

“In contrast, 32 of the remaining 69 volunteers developed AMS, representing 46.4% (32/69). Pearson χ^2^ test showed a statistically significant difference between the two groups (χ^2^=5.58, P =0.018). Mutation carriers had an 82% lower risk of developing AMS (OR = 0.18, 95% CI: 0.04–0.85).”

Due to potential ambiguities in the original expression, we have revised the fifth sentence (“Within 3 hours…”) and the ninth sentence (“All volunteers completed…”).

A correction has been made to the section **Materials and methods**, *High-Altitude Experiments*, Paragraph 1:

“After resting for several hours at 2800 m, all volunteers were transported by bus to the final destination (4000 m) within 3 hours, where monitoring was terminated. [ … ] Upon arrival at 4000 m, a treadmill exercise test was conducted as the first procedure.”

The original version of this article has been updated.

